# Case Report and Review of the Literature: Fatal Reversible Cerebral Vasoconstriction Syndrome

**DOI:** 10.3389/fneur.2021.589062

**Published:** 2021-02-15

**Authors:** Gautier Breville, Amelie Bailly, Loraine Fisch, Zsolt Kulcsar, Deborah Pugin, Emmanuel Carrera

**Affiliations:** ^1^Division of Neurology, Department of Clinical Neurosciences, Geneva University Hospitals, Geneva, Switzerland; ^2^Intensive Care Unit, Department of APSI, Geneva University Hospitals, Geneva, Switzerland; ^3^Neurovascular Unit, Department of Neurology, Groupement Hospitalier de l'Ouest Lémanique, Nyon, Switzerland; ^4^Division of Interventional Neuroradiology, Diagnostic Department, Zurich University Hospitals, Zurich, Switzerland

**Keywords:** reversible cerebral vasoconstriction syndrome, case report, stroke, angioplasty, calcium channel blocker, literature review of fatal outcome

## Abstract

**Background:** A fatal outcome occurs in 2% of patients with Reversible Cerebral Vasoconstriction Syndrome (RCVS). Due to its rarity, guidelines for the management of the most severe forms of RCVS are lacking.

**Case presentation:** Here, we describe the case of a 55 year-old woman who died from complications of RCVS and reviewed patients with fatal outcome reported in the literature. In our patient, the first episode of neurological deterioration was preceded by an increase of cerebral blood flow velocities assessed with transcranial Doppler. A fatal evolution could not be prevented despite therapeutic escalation consisting of multiple non-invasive and invasive treatments including cervical sympathetic bloc and continuous arterial infusion of nimodipine at the site of severe vasoconstriction.

**Conclusion:** This case and the review of literature illustrate the challenges in the management of patients with severe RCVS. We describe here how monitoring of cerebral blood flow might help anticipate clinical worsening at the beginning of the disease and propose novel invasive and non-invasive therapeutic strategies based on monitoring of neurophysiological parameters.

## Introduction

The typical presentation of reversible cerebral vasoconstriction syndrome (RCVS) is characterized by recurrent thunderclap headaches with or without acute neurological symptoms. Favorable outcome is reported in most case (1), although ischemic and hemorrhagic strokes can occur, leading to death in up to 2% of the cases (2). The therapeutic approach of patients suffering from severe RCVS remains a challenge for clinicians.

Here, we report the case of a patient with severe RCVS and fatal outcome and review the literature for similar patients. We investigated how monitoring of cerebral blood flow can help anticipate clinical worsening and propose novel invasive and non-invasive therapeutic strategies based on their impact neurophysiological data.

## Methods

The systematic review of case reports/series of fatal RCVS used “pubmed” search to retrieve publications of patients with RCVS leading to death. Key words used included “reversible cerebral vasoconstriction syndrome” AND “death” (articles selected for the review/articles found on pubmed: 2/18); AND “fatal” (4/11); AND “fulminant” (5/8) AND “died” (5/25); AND “decease” (0/24); AND “poor outcome” (4/10). We excluded the four fatal cases mentioned in the retrospective observational study published by Katz et al. (3) (59 patients) because of the lack of information, especially regarding treatments.

## Case Presentation

A 55-year-old woman experienced unusual recurrent episodes of thunderclap headaches (Day 0). The waxing and waning evolution caused the patient to trivialize the situation at initial stages. She was treated for bipolar disorder with aripiprazole and venlafaxine. The patient was smoking tetrahydrocannabinol (THC) regularly and benefited from long-term opioid substitution therapy (buprenorphine). Seven days before admission, she started using an over-the-counter nasal decongestant spray, including tuaminoheptane, a sympathomimetic vasoconstrictor.

**Figure 1:**
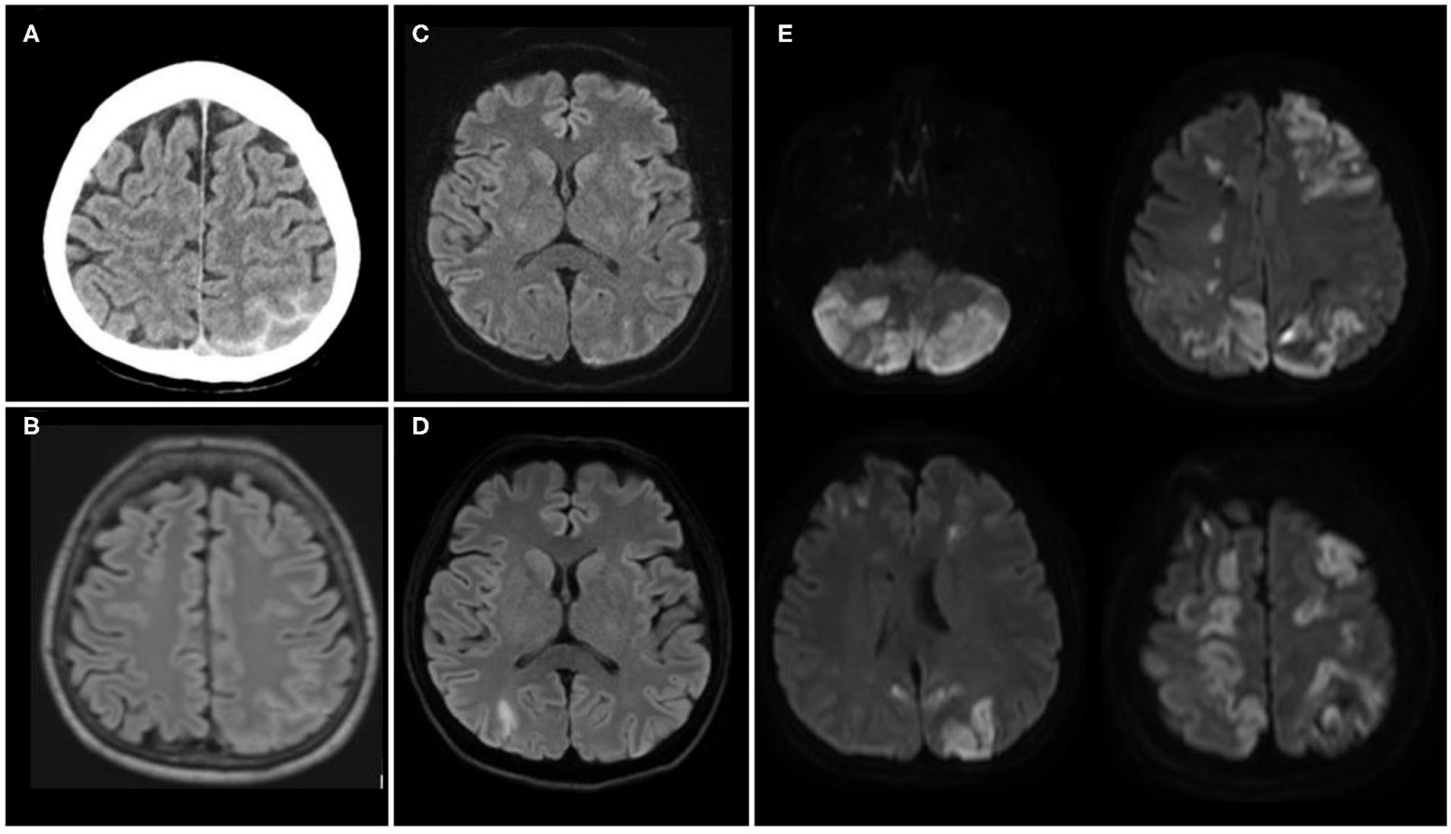
Illustration of radiological brain lesions over time. **(A)** Left convexity subarachnoid hemorrhage (SAH) on Day 4 (non-contrast CT). **(B)** Brain parenchyma without damage next to the left convexity SAH on Day 4 [MRI, T2 Fluid Attenuated Inversion Recovery (FLAIR)]. **(C)** Brain parenchyma without damage on Day 4 [MRI, Diffusion Weighted Imaging (DWI)]. **(D)** Right watershed infarct at the border zone between right middle and posterior cerebral arteries on Day 11 at the time of first neurological deterioration (MRI, DWI). **(E)** Multiple hemodynamic strokes in supratentorial and infratentorial regions on Day 18 (MRI, DWI).

**Figure 2:**
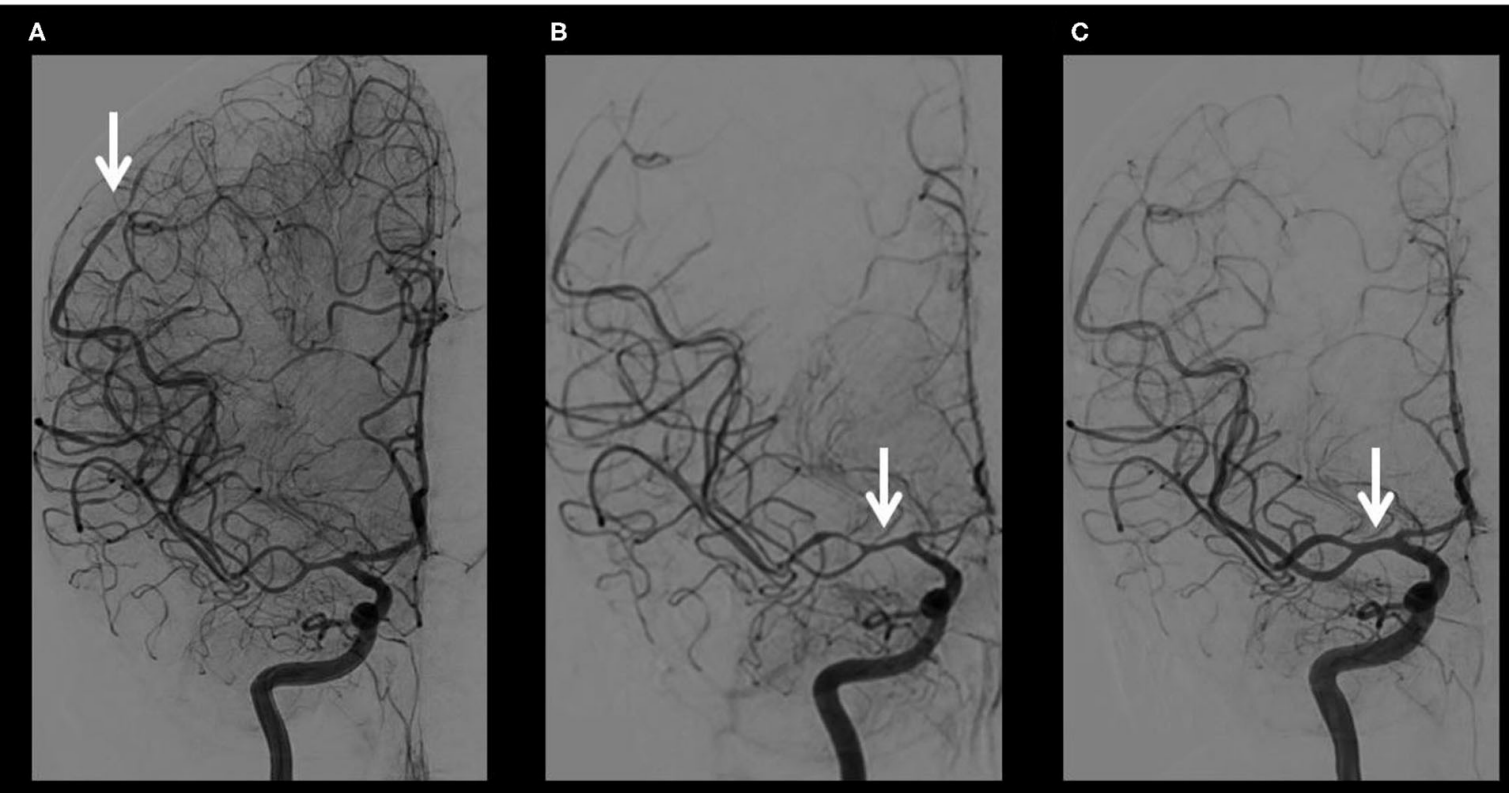
Illustration of cerebral digital subtraction angiographies (DSA) over time and response to treatments. **(A)** Cerebral Digital Subtraction Angiography (DSA) on Day 5 evidencing widespread arterial vasoconstrictions predominantly in brain distality (white arrow). **(B)** Cerebral DSA on Day 12 showing worsened distal and proximal (white arrow) arteries stenoses before intraarterial treatments. **(C)** Cerebral DSA on Day 12 showing distal and proximal (white arrow) arteries stenoses resolution after intraarterial injection of nimodipine and balloon angioplasty on proximal stenosis.

**Figure 3:**
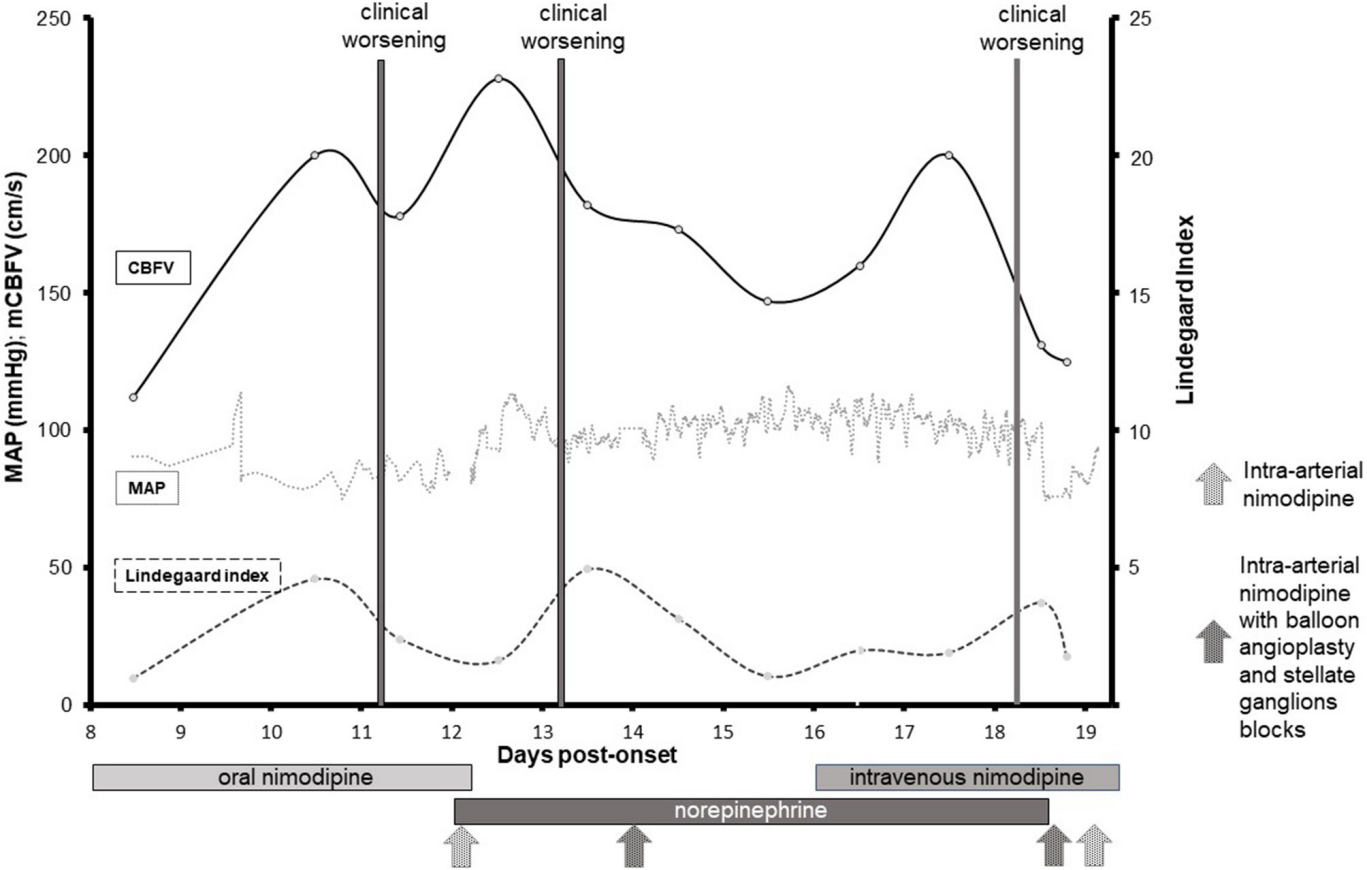
Evolution of physiological measures and effect of treatments. Evolution of mean arterial pressure (MAP), mean cerebral blood flow velocity (mCBFV), and Lindegaard index from Day 8 to 19. The three gray vertical bars indicate the episodes of clinical deterioration (Day 11, 13, 18).

On Day 4, she decided to consult at the emergency room of the closest hospital. Brain imaging by contrast CT (Day 4) and MRI (Day 4) showed a left parieto-occipital subarachnoid hemorrhage (SAH) (Figure 1). Cerebral vessels imaging by digital subtraction angiography (DSA, Day 5) evidenced widespread vasoconstrictions in proximal and distal arteries (Figure 2). That was not seen on initial MRA. Cerebrospinal fluid (CSF) analysis revealed high red blood cells (11'200/mm3), moderate elevation of protein level (0.94 g/L; <0.45) and normal white blood cells (0/mm3; <5). According to the international classification of headache disorders (ICHD3), we assumed the diagnosis of RCVS (4) and a calcium channel blocker therapy was started (nimodipine 30 mg tid orally). On Day 10, because of intracranial vasoconstrictions on transcranial Doppler (TCD) (mean cerebral blood flow velocities (mCBFV) in the right MCA at 200 cm/s and Lindegaard Index at 4.76), nimodipine was increased to 60 mg q.4h (Figure 3). On Day 11, the patient presented a left homonymous lateral hemianopsia (HLH) and fluctuant left upper limb paresis. Brain MRI showed acute border zone ischemic strokes (Figure 1).

Because of a decrease in mean arterial pressure (MAP) below 70 mmHg following nimodipine increase, the patient was admitted to the intensive care unit (Day 12). The therapeutic strategy aimed at increasing cerebral blood flow (CBF) with initiation of norepinephrine (MAP target 90–110 mmHg), and discontinuation of calcium channel blocker therapy to avoid vasodilatation with potential hypoperfusion. Elevation of mCBFV to 209 cm/s (Lindegaard Index 2.9) was found on Day 12, one day before new clinical worsening. Widespread acute bilateral ischemic lesions were found on NCCT and a second DSA revealed worsened bilateral proximal and distal arteries stenoses (Figure 2). Balloon angioplasty of the proximal MCA was performed, as well as intraarterial nimodipine administration, locally (Figure 2) and in both internal carotid arteries (Day 12, 14, 18, and 19), resulting in cerebral vasodilatation which however did not last after infusion cessation. Stellate ganglia bloc (level C6–C7) was performed on Day 14 (bupivacaine 0.5) with transient effects on vasoconstriction. On Day 17, persistent elevated mCBFV (202 cm/s) without significant change in Lindegaard Index (2.0) was observed hours before new clinical worsening. On Day 22, multiple cerebral ischemic and hemorrhagic strokes, as well as bi-hemispheric edema, were seen on NCCT. Because of the severity of the clinical examination (coma), the extent of brain lesions and limited additional therapies, a withholding therapy was considered after discussion with her relatives. The patient died on Day 23 due to brain herniation.

## Ethics

Written informed consent was obtained from the individual's legal next of kin for the publication of any potentially identifiable images or data included in this article.

## Discussion

Nasal vasoconstrictors, chronic use of THC and selective serotonin recapture inhibitor are all identified as potential RCVS triggers (5). Female gender and age of the patient at onset are also independent risk factors for RCVS (2). RCVS typically begins with distal arteriolar vasoconstriction with a centripetal course leading to normal and falsely reassuring TCD examination in the first days after onset. In our case, the monitoring of CBFV identified the development of proximal vasoconstriction at least several hours before neurological deterioration (Figure 3). The role of TCD to monitor cerebral vasospasm or delayed cerebral ischemia after aneurysmal SAH is currently debated (6, 7) but low or very high MCA CBFV (<120 or >200 cm/s) seem to reliably predict the absence or presence of clinically significant vasospasm (8, 9). The literature is more limited concerning the predictive value of TCD in patients with RCVS, which prevent to draw a conclusion about the role of TCD, especially because, in our case, none of the interventions based on TCD resulted in a positive outcome. Only a small case series of RCVS patients have been reported showing an increased CBFV in at least one major cerebral blood vessel (middle, anterior and posterior cerebral arteries, vertebral arteries, basilar artery) by initial TCD after RCVS diagnosis, and a peak MCA velocity reached 3–4 weeks after the onset of thunderclap headache (10). Chen et al. (11) demonstrated that MCA CBFV maintained a high plateau at the meantime of headache remission (Day 22) and remained abnormal even 10 days after headache resolution. TCD monitoring of RCVS patients may be difficult because the physiopathology of RCVS consists of the association of vasodilatation and vasoconstriction. Consequently, normal TCD could not rule out further RCVS aggravation. In our case, the elevation of CBFV preceded the development of proximal vasoconstriction at least several hours before the first ischemic stroke-related neurological deterioration (Figure 3). The prediction of the two other neurological deteriorations is not as clear possibly because TCD examinations were performed while the patient was admitted in the ICU under several vasoactive treatments (norepinephrine infusion, calcium channel blockers). The absence of continuous TCD monitoring limits further interpretations while RCVS is a dynamic process with permanent arterial caliber changes.

This case also illustrates the difficulties in treating the most severe cases of RCVS that might not be as “reversible” as this adjective suggests. As recommended by experts, we started with nimodipine therapy, initially orally then intravenously and finally using intraarterial *in situ* administration. Cerebral vasodilatation was demonstrated during local nimodipine administration (Figure 2), but the effects did not last after drug cessation. No validated guideline exists regarding doses of nimodipine. Even if the effect of nimodipine seems to be specific mainly to intracerebral arteries at low doses, higher intravenous doses also have a significant impact on systemic arterial blood pressure with a risk of developing cerebral hypoperfusion in patients with widespread vasospasms. Combination of systemic and local mechanisms may explain border zone ischemia that occurred in our patient when MAP dropped following increased doses of nimodipine. This therapy is more harmful at a later stage given the risk of bleeding in the already infarcted cerebral tissue due to reperfusion syndrome. As a second-line therapy, to increase CBF, hypertensive therapy was initiated with high doses of norepinephrine. Despite successful maintenance of MAP in the target range, clinical fluctuations were observed. A deleterious effect of norepinephrine cannot be ruled out as a cerebral vasoactive effect has been described although the vasoconstrictive effect of norepinephrine is mainly at the splanchnic and cutaneous levels (12). As a third line therapy, a balloon angioplasty and a bilateral stellate block (bupivacaine 0.5%) by analogy with previous reports in patients with vasospasm following SAH (13) were not associated with significant long term effect based on TCD monitoring. In the absence of evidence-based guidelines for this extremely rare situation, all the unsuccessful interventions failed to save patient life and raised the question of iatrogenicity. More data are needed to determine the benefits and the risks of this approach in severe cases of RCVS.

In the literature, death in the context of RCVS has been rarely reported (5). Table 1 summarizes previously described cases of RCVS with fatal outcome (14–27). RCVS occurrence peaks take place around 42-year-old, and the syndrome is more common in women than in men (5). Our fatal cases series seems to respect these data with a mean age of 41-year-old and 19 women out of 21 reported patients. Several potential causes of RCVS have been reported in patients with fatal RCVS that are similar those triggering more benign forms of RCVS (1), and these commonly known factors also trigger fatal RCVS (anti-depressive treatments, post-partum, THC consumption, nasal decongestant). Many of these cases (13/21) received steroids (methylprednisolone, prednisone, fludrocortisone) without positive effect. This observation is in line with recent comments suggesting glucocorticoid exposure as a contributing factor to worsening in RCVS (28, 29). Oral calcium channel blockers administration was initiated orally (but not intravenously) in 14/21 patients without favorable outcome. Second line therapies intended to improve global cerebral perfusion (norepinephrine or vasopressin to increase MAP) and third line interventional therapies (balloon angioplasty, stellate block) have only been used in, respectively, six and four cases out of 21, which limit definitive conclusions about their efficacy. Treatments reducing intracerebral pressure (osmotic therapy, hypothermia, fentanyl, and propofol) have been employed in rescue situations in patients with life-threatening cerebral complications (ischemic stroke, hemorrhagic stroke, cerebral edema).

**Table 1:** Baseline characteristics and treatments in our case and in the literature.

**Population, disease and treatments characteristics**	**Case**	**Buckle et al. (14)**	**Geraghty et al. (15)**	**Williams et al. (16)**	**Marshall et al. (17)**	**Singhal et al. (18)**	**Fugate et al. (19)**	**Fugate et al. (19)**	**Fugate et al. (19)**	**Fugate et al. (19)**	**John et al. (20)**	**Robert et al. (21)**	**Robert et al. (21)**	**Thydén et al. (22)**	**Sabzwari et al. (23)**	**Suchdev et al. (24)**	**Holanda Pena et al. (25)**	**Kunchok et al. (26)**	**Kunchok et al. (26)**	**Kunchok et al. (26)**	**Kunchok et al. (26)**
Age	55	16	27	40	57	36	33	32	30	15	45	68	53	58	32	25	36	61	24	63	55
Gender	F	F	F	F	F	F	F	F	F	F	F	F	F	M	F	F	F	F	M	F	F
Trigger	Venlafaxine, THC, tuaminoheptane	None	PP	PP	None	PP	PP	PP	PP	PP	Escitalopram,THC	None	THC	None	PP	None	PP	Escitalopram	THC	Nasal decongestant	Sertraline
Death delay after clinical onset (days)	23	2	21	14	25	28	25	18	14	7	21	4	13	7	12	21	36	28	21	14	42
**First line therapies**																					
Nimodipine	X					X	X	X				X	X	X	X	X	X	X	X		
Nicardipine						X					X				X						
Verapamil			X				X	X			X				X						
**Second line therapies**																					
Norepinephrin	X	X				X									X						
Vasopressin							X	X													
**Third line therapies**																					
Balloon Angioplasty	X						X					X	X								
Stellate bloc	X																				
**Other used therapies**																					
Steroids: MP/P/fludrocort			X	X	X	X	X	X			X					X	X	X	X	X	X
Cyclophosphamide				X																X	X
Rituximab																					X
Immunoglobulins																	X				
Labetalol										X											
Hypertonic therapy				X		X			X	X					X	X					
Hypothermia									X							X					
Fentanyl+propofol																X					
Magnesium				X		X	X														
Epoprostenol														X							

*THC, tetrahydrocannabinol; F, female; M, male; PP, post-partum; fludrocort, fludrocortisone; MP, methylprednisolone; P, prednisone*.

Our case has several limitations. First, because of the rapid deterioration of the patient's condition, we combined several treatments that may have acted synergistically or with opposite mechanisms. Therefore, the individual effect of each treatment is challenging to assess. Second, none of our therapeutic strategies resulted in a favorable outcome. We cannot rule out that some of these treatments may have been harmful to the patient.

This case illustrates the challenges in monitoring and treating patients with severe RCVS. Further studies have to be achieved to determine the role of TCD, especially at an early stage of the disease. In particular, continuous monitoring of CBFV should be evaluated in patients with RCVS. Despite the fatal outcome in our patients, we used several invasive therapies that may be effective in similar patients if initiated early enough.

## Data Availability Statement

The raw data supporting the conclusions of this article will be made available by the authors, without undue reservation.

## Ethics Statement

Written informed consent was obtained from the individual's legal next of kin for the publication of any potentially identifiable images or data included in this article.

## Author Contributions

GB and EC designed this article. GB drafted the first manuscript. AB, LF, ZK, DP, and EC contributed to interpretations of clinical and radiological details. All authors read and approved the final manuscript.

## Conflict of Interest

The authors declare that the research was conducted in the absence of any commercial or financial relationships that could be construed as a potential conflict of interest.

## Abbreviations

CBF: cerebral blood flow
CBFV: cerebral blood flow velocities
CSF: cerebrospinal fluid
DSA: digital subtraction angiography
HLH: homonymous lateral hemianopsia
MAP: mean arterial pressure
MCA: middle cerebral artery
NCCT: non-contrast CT scanner
RCVS: reversible cerebral vasoconstriction syndrome
SAH: subarachnoid hemorrhage
TCD: transcranial Doppler
THC: tetrahydrocannabinol.
